# Usefulness of serum creatinine and cystatin C ratio as a screening tool for predicting prognosis in patients with pancreatic cancer

**DOI:** 10.1002/ags3.12671

**Published:** 2023-04-25

**Authors:** Mariko Tsukagoshi, Akira Watanabe, Kenichiro Araki, Norihiro Ishii, Kei Hagiwara, Kouki Hoshino, Ryo Muranushi, Norifumi Harimoto, Makiko Takizawa, Ken Shirabe

**Affiliations:** ^1^ Division of Hepatobiliary and Pancreatic Surgery, Department of General Surgical Science Gunma University Graduate School of Medicine Maebashi Gunma Japan; ^2^ Department of Healthcare Quality and Safety Gunma University Graduate School of Medicine Maebashi Gunma Japan

**Keywords:** creatinine, cystatin C, pancreatic cancer, prognosis, sarcopenia

## Abstract

**Aim:**

This study aimed to evaluate the usefulness of the serum creatinine/cystatin C (Cr/CysC) ratio as a prognostic factor after pancreatic surgery in patients with pancreatic cancer.

**Methods:**

We retrospectively analyzed the data of 88 patients with pancreatic ductal carcinoma who underwent pancreatic surgery from January 2017 to December 2020. CysC measured from frozen serum samples and circulating Cr levels were used to calculate the Cr/CysC ratio. The cutoff value of the Cr/CysC ratio was determined using receiver operating characteristic curves. Cox proportional hazards model analysis and survival curves were applied to identify the prognostic factors.

**Results:**

The optimal cutoff value of the Cr/CysC ratio for predicting mortality after surgery was 1.05. This study included 20 (22.7%) and 68 (77.3%) patients with high and low Cr/CysC ratios, respectively. The low Cr/CysC ratio was significantly associated with female sex (*p* = 0.020) and higher levels of C‐reactive protein (*p* = 0.020). The postoperative length of stay was significantly longer in patients with low Cr/CysC rates (*p* = 0.044). Patients with low Cr/CysC ratio showed poorer prognosis in relapse‐free survival (hazard ratio [HR] = 3.33; 95% confidence interval [CI]: 1.54–4.20; *p* = 0.002) and overall survival (HR = 2.52, 95% CI: 1.04–6.10, *p* = 0.041), respectively, which were significantly worse than in those with high Cr/CysC ratios (*p* = 0.003 and 0.049, respectively).

**Conclusion:**

The Cr/CysC ratio could be a useful screening tool for predicting the prognosis of patients with pancreatic ductal carcinoma undergoing pancreatic surgery.

## Graphical abstract

This study evaluated the usefulness of the serum creatinine/cystatin C (Cr/CysC) ratio as a prognostic factor after pancreatic surgery in patients with pancreatic cancer. The present study revealed the low Cr/CysC ratio was an independent prognostic indicator of poor relapse‐free survival and overall survival after pancreatic surgery. These findings suggest that the preoperative Cr/CysC ratio could be a new simple prognostic indicator in patients with pancreatic ductal carcinoma postoperatively.

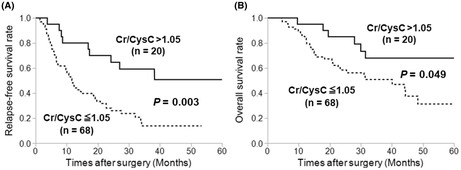

## 1 INTRODUCTION

Pancreatic cancer is one of the most lethal malignancies with a low 5‐year relative survival rate of only 11%.^1^ Surgical resection is the only potentially curative treatment option for patients with pancreatic cancer. Postoperative recurrence rates are high and most patients fail to achieve long‐term survival despite advances in surgical procedures.^2^ Thus, appropriate and quantitative evaluation is required to select candidates for surgical resection and identify patients at high risk of recurrence.

Sarcopenia, which is a loss of skeletal muscle mass and strength, was identified as a poor prognostic predictor in patients with pancreatic cancer undergoing pancreatic surgery.^3^ Moreover, our previously published study revealed that preoperative skeletal muscle loss and octogenarian status predicted the failure of S‐1 adjuvant chemotherapy completion in patients with pancreatic cancer undergoing pancreatic surgery.^4^ The currently revised international consensus suggests low muscle strength, which was identified using grip strength and chair stand test, as the primary parameter of sarcopenia.^5^ The European Working Group on Sarcopenia in Older People (EWGSOP2) algorithm recommended the next step of muscle quantity or quality evaluation as bioelectrical impedance analysis (BIA), dual‐energy X‐ray absorptiometry, magnetic resonance imaging, or computed tomography (CT) for sarcopenia diagnosis.^5^ Moreover, the severity of sarcopenia is identified by physical performance measures, such as gait speed. These diagnostic methods are costly, time‐consuming, and not widely available in clinical practice. Therefore, simple and noninvasively obtained biomarkers are needed to diagnose sarcopenia and predict the prognosis of patients with pancreatic cancer.

Serum creatinine (Cr) is the most common clinical marker of the glomerular filtration rate (GFR) and renal function.^6^ In general, serum Cr is affected by skeletal muscle mass because it is an endogenous product of muscle catabolism. Therefore, the serum Cr level has been used as a useful approximation of muscle mass.^7^ Meanwhile, cystatin C (CysC) is a small protein derived from all nucleated cells and is reabsorbed and completely catabolized at the proximal tubule. Moreover, its production is less affected by muscle mass. Thus, CysC is considered a more reliable marker of renal function than serum Cr.^8^ Recently, several reports revealed that the Cre/CysC ratio, calculated as the serum Cr divided by the serum CysC, was a biomarker for low skeletal muscle mass and sarcopenia in different populations, including patients with type 2 diabetes,^9^ non‐dialysis chronic kidney disease,^10^ and the elderly.^11^ A most recent study revealed the serum Cr/CysC ratio as a surrogate marker and prognostic indicator in patients with esophageal and gastric cancer.^12^, ^13^ However, the value of the Cr/CysC ratio in patients with pancreatic cancer was not reported. Therefore, this study aimed to investigate the association between the Cr/CysC ratio and the prognosis in patients with pancreatic cancer after pancreatic surgery.

## 2 METHODS

### 2.1 Study design and participants

This study retrospectively analyzed 88 patients with pancreatic ductal carcinoma (PDAC) who underwent pancreatic resection from January 2017 to December 2020 in the Department of Hepatobiliary and Pancreatic Surgery at Gunma University Hospital. The study was approved by the Ethics Committee of the study hospital (HS2019‐306) and met the institutional guidelines and the Declaration of Helsinki. We excluded patients with intraductal papillary mucinous carcinoma.

### 2.2 Treatment and data collection

Demographic and clinical characteristics and treatment‐related details of all patients were collected from the medical records. Surgical procedures were performed according to institutional policies and institutional cancer board recommendations. Postoperative complications in 30 days were recorded and scored according to the Clavien–Dindo classification.^14^ Resected tumors were classified according to the TNM Classification of Malignant Tumors of the Union for International Cancer Control (8th version).

Relapse‐free survival (RFS) is the period from the date of surgery until the date of documented disease progression or all‐cause death. Overall survival (OS) is the period from the date of surgery to the date of all‐cause death.

### 2.3 Laboratory measurements

Frozen serum samples collected on the morning of surgery were used to measure CysC. Serum CysC levels were quantified using latex agglutination turbidimetric immunoassay (N‐assay LA CysC NITTOBO; Nitto Boseki Co., Ltd.) at the central laboratory of Gunma University Hospital. Circulating Cr levels were measured by routine blood tests on the day before surgery. Differences in sample collection timings were within 24 h. According to the literature, the Cr/CysC ratio was calculated as follows: Cr/CysC ratio = {(serum Cr (mg/dL))/serum CysC (mg/L)} × 100.

### 2.4 Skeletal muscle mass and strength assessment

The area of skeletal muscle mass at the inferior aspect of the third lumbar vertebra (L3) was measured using CT images obtained within 30 days preoperatively. A trained investigator blinded to all anthropometric and surgical characteristics identified and measured the skeletal muscle area using a dedicated processing system, the volume analyzer SYNAPSE VINCENT (Fujifilm Medical, Tokyo, Japan), to minimize measurement bias. The cross‐sectional area (cm^2^) of the skeletal muscle in the L3 region was measured by rough manual outlining on CT images, and the total cross‐sectional area of the segmented tissue was automatically calculated. Muscle areas computed from each image were normalized as follows: Skeletal muscle mass index (SMI) = cross‐sectional areas of the skeletal muscle mass in the L3 region/height^2^ (cm^2^/m^2^). The cutoff values for the SMI (cm^2^/m^2^) were 42 cm^2^/m^2^ for males and 38 cm^2^/m^2^ for females, as proposed by the Japan Society of Hepatology.^15^

Muscle strength was identified using hand grip strength. The cutoff values for hand grip strength were 28 kg for males and 18 kg for females, according to the Asian Working Group for Sarcopenia.^16^ Sarcopenia was defined as low muscle strength (<28 kg for males and <18 kg for females), plus low SMI (<42 cm^2^/m^2^ for males and <38 cm^2^/m^2^ for females).

### 2.5 Evaluation of inflammatory and nutritional factors

We evaluated prognostic indicators based on inflammatory and nutritional factors, including neutrophil‐to‐lymphocyte ratio (NLR) and prognostic nutritional index (PNI). PNI was calculated as 10 × serum albumin (g/dL) + 0.005 × total lymphocyte count (/mm^3^).^17^

### 2.6 Follow‐up

All patients were examined every 3 months by tumor marker and CT scan for recurrence after discharge. Recurrent PDAC was treated by chemotherapy, radiotherapy, or heavy ion radiotherapy depending on the recurrence situation.

### 2.7 Statistical analysis

Categorical variables were assessed using the chi‐square test or Fisher's exact test, as appropriate. Survival curves were estimated using the Kaplan–Meier method, and the log‐rank test was used to analyze the differences between the curves. Cox proportional hazards model analysis was performed in univariate and multivariate analyses of prognostic factors. All statistical analyses were performed using the JMP Pro 14 statistical software (SAS Institute, Cary, NC, USA). A *p*‐value of <0.05 was considered statistically significant.

## 3 RESULTS

### 3.1 Clinical characteristics of patients in the two groups classified by the Cr/CysC ratio

The receiver operating characteristic curve (ROC) was plotted to determine the optimum serum Cr/CysC ratio for predicting mortality after surgery in patients with PDAC. The best cutoff value of the Cr/CysC ratio for mortality was 1.05 (area under the curve = 0.54) (Figure 3). Based on the cutoff value, the sensitivity, specificity, positive predictive value, and negative predictive value were determined to be 85.4%, 30.0%, 51,5%, and 70.0%, respectively.

A comparison of clinicopathological characteristics of patients with high (>1.05) and low Cr/CysC ratios (≦1.05) is summarized in Table 1. Of the 88 patients, 20 (22.7%) and 68 (77.3%) patients had high and low Cr/CysC ratios. Compared to high Cr/CysC, low Cr/CysC was significantly associated with female sex (*p* = 0.020) and higher C‐reactive protein (CRP) levels (*p* = 0.020). The postoperative length of stay was significantly longer in patients with low Cr/CysC ratios (*p* = 0.044), but without a significant difference in Clavien–Dindo grade ≥3 of postoperative complications or completion of S‐1 adjuvant chemotherapy.

**TABLE 1:** Comparison of the clinicopathological factors between the two groups classified by the Cr/CysC ratio.

Variables	Cr/CysC ratio		*p*‐value
	Low (*n* = 68)	High (*n* = 20)	
Age (years)	71 (46–85)	75 (42–88)	0.113
Male	26 (38.2%)	14 (70.0%)	0.020*
ASA‐PS score ≥3	10 (14.7%)	7 (35.0%)	0.057
Body mass index (kg/m^2^)	21.1 (15.9–33.0)	21.0 (17.4–33.6)	0.992
Skeletal muscle area in the L3 region (cm^2^)			
Male	10 110 (5560–15 287)	11 072 (8498–15 657)	0.183
Female	7194 (4953–11 792)	7380 (6265–9566)	0.632
Skeletal muscle mass index (cm^2^/m^2^)			
Male	40.2 (22.3–57.5)	40.7 (34.5–58.9)	0.238
Female	32.2 (21.4–50.7)	30.9 (25.7–41.4)	0.837
Hand grip strength (kg)			
Male	32.5 (20.3–48.8)	35.9 (31.1–41.6)	0.279
Female	21.2 (11.3–30.1)	21.2 (18.8–25.8)	0.906
Resectability			0.501
Resectable	58 (85.3%)	19 (95%)	
Borderline	9 (13.2%)	1 (5%)	
Unresectable	1 (1.5%)	0	
Preoperative chemotherapy	14 (20.6%)	1 (5.0%)	0.174
nab‐Paclitaxel+Gemcitabine	7 (10.3%)	1 (5.0%)	
Gemcitabine+S‐1	7 (10.3%)	0	
Parameters			
Albumin (g/dL)	3.7 (2.7–4.5)	3.9 (3.4–4.4)	0.092
Hemoglobin (g/dL)	11.8 (6.9–15.3)	12.2 (8.6–14.8)	0.282
Lymphocytes (/μL)	1320 (550–2740)	1285 (670–2520)	0.728
CRP (mg/dl)	0.10 (0.01–4.47)	0.05 (0.01–1016)	0.020*
Zn (μg/dL)	81 (55–105)	71 (61–107)	0.634
CA19‐9 (U/mL)	58 (2–22 472)	68 (2–1292)	0.650
DUPAN‐2 (U/mL)	122 (25–16 000)	109 (25–1933)	0.652
Span‐1 (U/mL)	40.3 (10.0–5114.7)	39.7 (10.0–1245.0)	0.562
Cystatin C (mg/L)	0.83 (0.50–1.75)	0.72 (0.56–0.98)	0.016*
Creatinine (mg/dL)	0.64 (0.40–1.29)	0.88 (0.62–1.14)	<0.001*
NLR	2.27 (0.70–11.83)	2.03 (0.95–9.00)	0.414
PNI	45.1 (32.2–52.4)	46.5 (39.2–56.5)	0.315
Tumor‐related factors			
Tumor size (cm)	3.3 (1.3–12.5)	3.1 (0–16.2)	0.676
Lymph node metastasis (+)	41 (30.3%)	11 (55.0%)	0.797
Operative procedures			
Pancteatoduodenectomy	45 (66.2%)	11 (55.0%)	0.431
Operative time (min)	459 (188–776)	411 (236–667)	0.171
Blood loss (ml)	315 (23–2964)	255 (10–2720)	0.527
Postoperative hospitalization (days)	25 (10–82)	16 (11–81)	0.044*
R1 resection	12 (17.7%)	4 (20.0%)	0.753
Complications (Clavien–Dindo grade ≥3)	21 (30.9%)	5 (25.0%)	0.782
Adjuvant S‐1 completion	30 (44.1%)	11 (55.0%)	0.450

*Note*: Data are expressed as median (interquartile range), or number of patient (%). *p value <0.05.

Abbreviations: ASA‐PS, American Society of Anesthesiologists physical status; Cr, creatinine; CysC, cystatin C; CRP, C‐reactive protein; NLR, neutrophil‐to‐lymphocyte ratio; PNI, prognostic nutritional index; Zn, Zinc.

### 3.2 Prognostic factors associated with RFS and OS

Univariate and multivariate analyses were performed to analyze factors considered for RFS in all patients (Table 2). Univariate analysis revealed CA19‐9 >37 U/mL, Span‐1 >30 U/mL, low Cr/CysC ratio, postoperative complications (Clavien–Dindo grade ≥1), lymph node metastasis, R1 resection, and S‐1 adjuvant chemotherapy for <6 months as significant factors for reduced RFS. Multivariate analysis revealed low Cr/CysC ratio (Hazard ratio [HR] = 3.33; 95% confidence interval [CI]: 1.54–4.20; *p* = 0.002), lymph node metastasis, R1 resection, and S‐1 adjuvant therapy for <6 months as independent prognostic indicators of poor RFS.

**TABLE 2:** Univariate and multivariate analyses of variables for RFS in patients with PDAC.

Variables	Univariate analysis			Multivariate analysis		
	Hazard ratio	95% CI	*p*	Hazard ratio	95% CI	*p*
Age ≥80 years	1.65	0.91–3.00	0.102			
Female	1.23	0.74–2.04	0.419			
PNI <45	1.28	0.77–2.11	0.343			
NLR ≥3	1.18	0.68–2.05	0.557			
CA19‐9 >37 (U/mL)	1.71	1.01–2.90	0.047*	1.17	0.50–2.74	0.722
DUPAN‐2 >150 (U/mL)	1.50	0.91–2.47	0.115			
Span‐1 >30 (U/mL)	2.04	1.21–3.44	0.008*	1.76	0.80–3.91	0.162
Low hand grip strength	1.09	0.55–2.16	0.813			
Low skeletal muscle mass index	0.96	0.54–1.71	0.884			
Low Cr/Cys ratio	3.13	1.52–6.46	0.002*	3.55	1.60–7.87	0.002*
Complications (Clavien–Dindo grade ≥3)	1.22	0.70–2.12	0.485			
Complications (Clavien–Dindo grade ≥1)	1.91	1.14–3.19	0.014*	1.17	0.65–1.13	0.601
Tumor size ≥30 mm	1.61	0.95–2.72	0.078			
Lymph node metastasis (+)	2.04	1.18–3.52	0.010*	1.75	0.98–3.13	0.057
R1 resection	1.93	1.07–3.47	0.029*	2.35	1.24–4.43	0.009*
S‐1 adjuvant therapy <6 months	2.77	1.65–4.67	<0.001*	3.45	1.99–5.98	<0.001*

Abbreviations: CI, confidence interval; Cr, creatinine; CysC, cystatin C; NLR, neutrophil‐to‐lymphocyte ratio; PDAC, pancreatic ductal carcinoma; PNI, prognostic nutritional index; RFS, relapse‐free survival.

* *p* value <0.05.

Univariate and multivariate analyses of factors for OS of all patients are shown in Table 3. Univariate analysis revealed age of ≥80 years, low Cr/CysC ratio, and S‐1 adjuvant chemotherapy for <6 months as significant factors for reduced OS. Multivariate analysis revealed low Cr/CysC ratio and S‐1 adjuvant therapy for <6 months as independent prognostic indicators of poor OS (HR = 2.52; 95% CI: 1.04–6.10; *p* = 0.041 and HR = 4.23; 95% CI, 1.96–9.11; *p* < 0.001, respectively).

**TABLE 3:** Univariate and multivariate analyses of variables for OS in patients with PDAC.

Variables	Univariate analysis			Multivariate analysis		
	Hazard ratio	95% CI	*p*	Hazard ratio	95% CI	*p*
Age ≥80 years	2.25	1.12–4.54	0.023*	0.99	0.47–2.09	0.974
Female	1.22	0.65–2.28	0.534			
PNI <45	1.07	0.57–2.00	0.832			
NLR ≥3	0.81	0.39–1.66	0.559			
CA19‐9 >37 (U/mL)	1.18	0.61–2.25	0.627			
DUPAN‐2 >150 (U/mL)	1.53	0.82–2.83	0.178			
Span‐1 >30 (U/mL)	1.37	0.72–2.61	0.334			
Low hand grip strength	1.75	0.75–4.10	0.195			
Low skeletal muscle mass index	1.45	0.68–3.10	0.331			
Low Cr/Cys ratio	2.51	1.05–6.01	0.038*	2.52	1.04–6.10	0.041*
Complications (Clavien–Dindo grade ≥3)	0.81	0.38–1.71	0.574			
Complications (Clavien–Dindo grade ≥1)	1.75	0.93–3.31	0.085			
Tumor size ≥30 mm	1.8	0.92–3.53	0.087			
Lymph node metastasis (+)	1.91	0.99–3.70	0.054			
R1 resection	1.61	0.78–3.30	0.196			
S‐1 adjuvant therapy <6 months	4.21	2.04–8.66	<0.001*	4.23	1.96–9.11	<0.001*

* *p* value <0.05.

Abbreviations: CI, confidence interval; Cr, creatinine; CysC, cystatin C; NLR, neutrophil‐to‐lymphocyte ratio; OS, overall survival; PDAC, pancreatic ductal carcinoma; PNI, prognostic nutritional index.

### 3.3 Association between Cr/CysC ratio and prognosis

**FIGURE 1:**
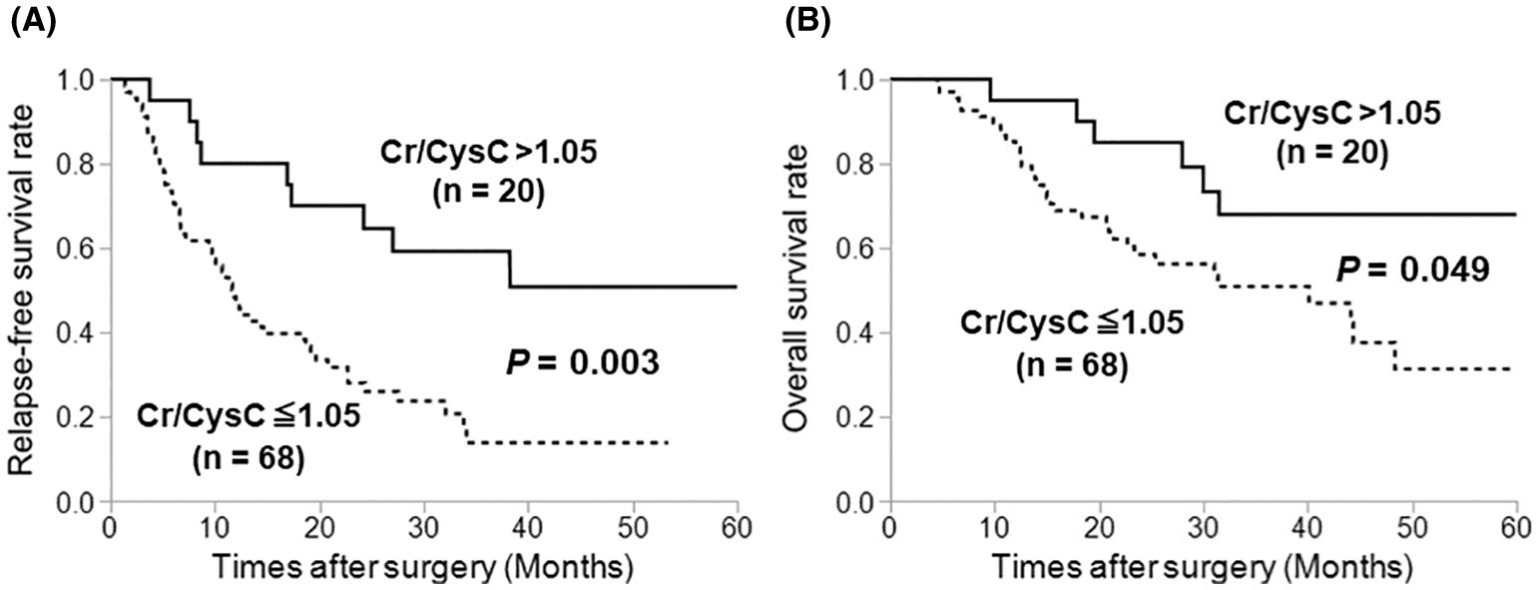
Kaplan–Meier curves for relapse‐free survival (A) and overall survival (B). Patients with low Cr/CysC ratios had significantly worse relapse‐free survival (*p* = 0.003) and overall survival (*p* = 0.049) rates than those with high Cr/CysC ratios. Abbreviations: Cr, creatinine; CysC, cystatin C.

The prognostic significance of the Cr/CysC ratio is shown in Figure 1. The median follow‐up period was 24.9 months (range, 4.5–66.7 months) in this study. Patients with low Cr/CysC ratios had significantly worse RFS (*p* = 0.003; Figure 1A) and OS (*p* = 0.049; Figure 1B) than those with high Cr/CysC ratios.

### 3.4 Correlation between Cr/CysC ratio and sarcopenia

**FIGURE 2:**
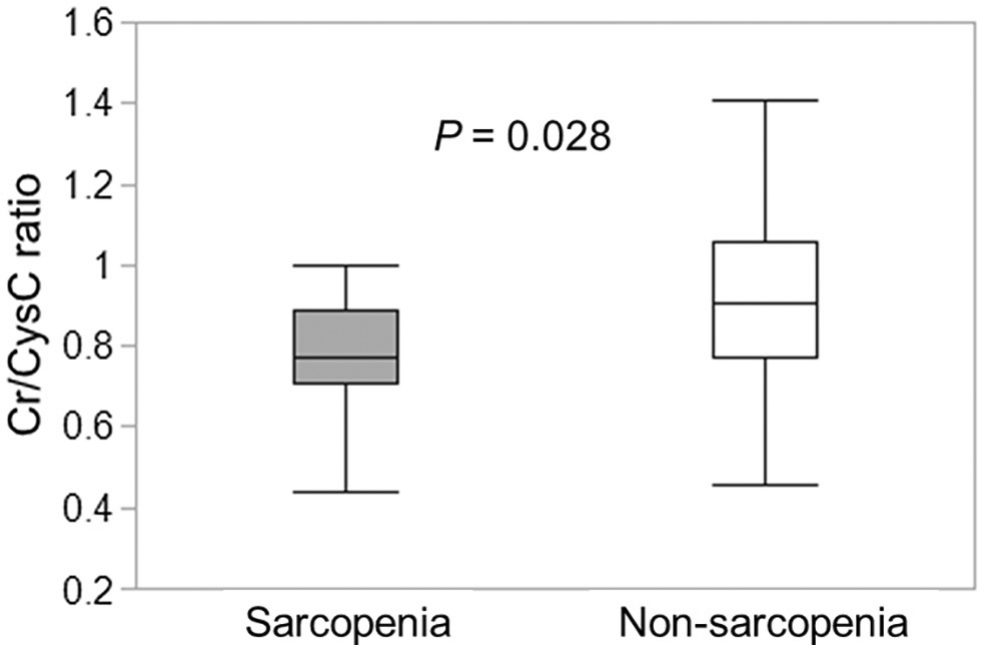
Correlation between the Cr/CysC ratio and sarcopenia. Cr/CysC ratio was significantly lower in patients with sarcopenia (*p* = 0.028). Abbreviations: Cr, creatinine; CysC, cystatin C.

We investigated the association between the Cr/CysC ratio and sarcopenia. Sarcopenia was defined as low muscle strength identified by hand grip strength, plus low SMI measured using CT. The Cr/CysC ratio was significantly lower in patients with sarcopenia (*p* = 0.028) (Figure 2).

**FIGURE 3:**
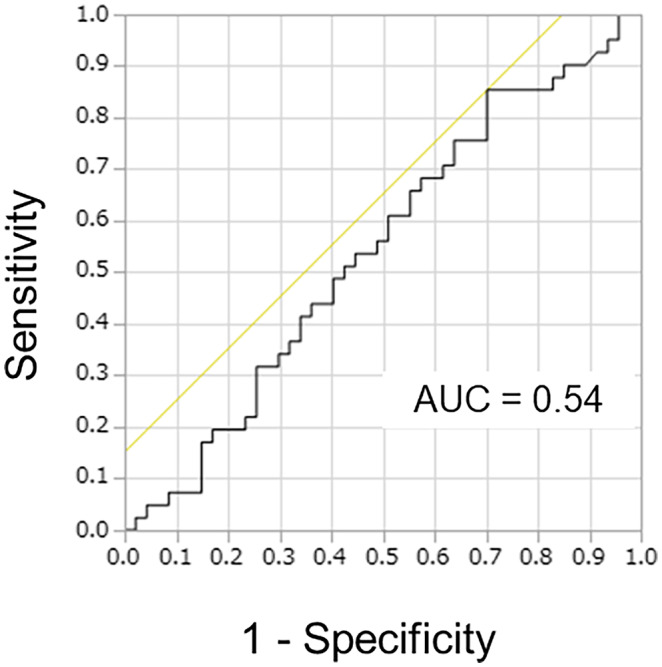
Receiver operating characteristic curve for the analysis of the serum Cr/CysC ratio for predicting postoperative mortality in patients with pancreatic ductal carcinoma. The best cutoff value of the Cr/CysC ratio for mortality was 1.05 (AUC = 0.54). Based on the cutoff value, the sensitivity, specificity, positive predictive value, and negative predictive value were 85.4%, 30.0%, 51.5%, and 70.0%, respectively. Abbreviations: AUC, area under the curve; Cr, creatinine; CysC, cystatin C.

## 4 DISCUSSION

The present study revealed an association between the Cr/CysC ratio and postoperative prognosis in patients with PDAC. The low Cr/CysC ratio was an independent prognostic indicator of poor RFS and OS after pancreatic surgery. These findings suggest that the preoperative Cr/CysC ratio could be a novel and uncomplicated screening tool for predicting the postoperative prognosis of patients with PDAC.

Sarcopenia was identified as a prognostic indicator for survival in patients with cancer and a predictor of postoperative complications; thus, it is a major clinical target in cancer treatment. Several techniques and parameters are used to diagnose sarcopenia. In clinical practice, SMI at the L3 level with CT remains the most commonly used technique, because CT is routinely performed for diagnosis, treatment evaluation, and follow‐up in cancer treatment.^18^ Our previous study used L3 SMI to define skeletal muscle loss, which revealed inadequate preoperative nutritional support and rehabilitation therapy as an independent risk factor for pancreatic fistula after PD in patients with skeletal muscle loss.^19^ In contrast to its high availability, the use of CT scans for monitoring body composition evaluation remains limited because of its high cost and radiation exposure. EWGSOP2 focused on low muscle strength as a key characteristic of sarcopenia.^5^ Measurement of handgrip strength is a simple and non‐invasive marker of muscle strength and has the predictive potential regarding postoperative complications and prognosis.^20^

Recently, the serum Cr/CysC ratio has been proposed to estimate muscle mass. A correlation between the Cr/CysC ratio and different muscle mass evaluation parameters was found in healthy individuals,^21^ critically ill patients,^22^ and patients with cancer.^11^, ^23^, ^24^ Ulmann et al.^24^ reported relatively good correlations between the Cr/CysC ratio and L3‐CT scan in patients with cancer, as well as better efficacy of the Cr/CysC ratio than that of BIA. Moreover, Tang et al.^25^ demonstrated a positive correlation between the Cr/CysC ratio and muscle mass by CT scan and handgrip strength in patients with advanced non‐small cell lung cancer (NSCLC). These reports have demonstrated that Cr/CysC ratio could be convenient and easy to use for muscle mass estimation in patients with cancer. Although CysC is a simple and reproducible biomarker, the application of CysC examination in medical insurance coverage remain a future concern.

Serum Cr and CysC are two compounds freely filtered by the kidney and were widely applied for renal function evaluation. CysC is produced by all nucleated cells at a constant rate, freely filtrated by the glomeruli, and completely catabolized in the proximal tubules.^26^ Serum CysC was superior to serum creatinine as a marker of GFR.^7^ Conversely, Cr levels may alter because its generation is not simply a product of muscle mass, but is influenced by muscle function, muscle composition, activity, diet, and health status.^26^ In our study, the SMI and hand grip strength were not significantly correlated with the Cr/CysC ratio, although the Cr/CysC ratio was significantly lower in patients with sarcopenia. Fu et al.^27^ revealed that the Cr/CysC ratio showed a relatively weak correlation with skeletal muscle mass (*r* = 0.299), indicating an imperfect correlation. SMI and handgrip strength were not prognostic indicators of poor RFS and OS in the present study. Jung et al.^28^ reported that the Cr/CysC ratio could also be an indicator of inflammatory status in addition to its correlation with muscle mass. Another possible explanation could be that CysC may reflect tumor burden. Therefore, this study suggested the association between the Cr/CysC ratio and muscle strength, nutritional status, and disease‐associated general condition in addition to skeletal muscle mass.

The ability of the Cr/CysC ratio to predict patient outcomes was investigated. Kashani et al.^22^ reported that Cr/CysC ratio was independently predictive of both hospital and 90‐day mortality among patients in the intensive care unit. Gao et al.^29^ revealed that the Cr/CysC ratio could effectively predict postoperative complications in patients with gastric cancer postoperatively. Zheng et al.^11^ reported a higher incidence of postoperative complications and poorer long‐term survival in patients with esophageal cancer with low Cr/CysC ratios. In a recent study, the Cr/CysC ratio revealed a significant association with mortality and hospitalization in patients with cancer regardless of age, sex, or cancer type.^28^ Moreover, the present study revealed the low Cr/CysC ratio as an independent prognostic indicator of poor RFS and OS in patients with PDAC postoperatively. Furthermore, the hazard ratio of Cr/CysC ratio was higher in RFS than in lymph node metastasis and R1 resection. This result indicated that patient factors such as Cr/CysC were more important for the prognosis of patients with postoperative PDAC than tumor factors, such as lymph node metastasis, and surgery‐related factors, such as R1 resection. These findings suggest that Cr/CysC ratio is a prognostic factor among patients with several cancers.

In recent years, several other factors based on serum Cr and CysC were reported. The association of eGFR based on CysC and hand grip strength was reported in older adults.^20^, ^30^ Additionally, previous reports showed that serum Cr × CysC‐based eGFR had a good correlation with low muscle mass or sarcopenia in patients with cancer.^23^, ^27^ Congruently, our previous study showed that high Cr × CysC‐based eGFR was significantly associated with low skeletal muscle mass and worse OS and RFS in patients undergoing hepatic resection for hepatocellular carcinoma.^31^ A study by Tang et al.^25^ demonstrated that the serum Cr/CysC ratio and serum Cr × CysC‐based GFR were positively correlated with muscle mass and handgrip strength in patients with advanced NSCLC. Additionally, the study revealed that the Cr/CysC ratio may outperform Cr × CysC‐based GFR in terms of predicting OS in patients with NSCLC. Further verification is required to determine the more reliable indicator.

The present study has several limitations. First, this single‐center retrospective observational study has a small sample size, which would have limited the generalizability of the results. Therefore, larger prospective studies are warranted to confirm and update the study conclusions. Second, we did not assess the physical performance (e.g., gait speed), which is one of the preferred criteria to determine the stage of sarcopenia. Third, the study has a selection bias; pancreatic surgery is often avoided in elderly patients because of high morbidity and mortality rates. Fourth, the timing of Cr and CysC measurements was not exactly simultaneous. Finally, the cutoff value of the Cr/CysC ratio was assessed using the area under the ROC curve (AUC–ROC), but the AUC–ROC was nearly equal to 0.5. This cutoff value had high sensitivity and high negative predictive value for mortality after surgery from PDAC, which means that patients with high Cr/CysC ratio are excluded from mortality risk. Conversely, patients with low Cr/CysC ratio are at risk and require further scrutiny. We sought the significance of the Cr/CysC ratio as a simple initial screening tool for predicting prognosis and assisting in subsequent treatment and intervention in patients with PDAC. However, the present study demonstrated the correlation between the Cr/CycS ratio and sarcopenia and the significance of the Cr/CysC ratio in prognostic value in patients with PDAC undergoing pancreatic surgery.

In conclusion, the Cr/CysC ratio evaluation could be an easy, objective, and replicable assessment tool and is a useful prognostic factor for RFS and OS in patients with PDAC. Hence, the Cr/CysC ratio might be the next gold standard for predicting cancer prognosis.

## AUTHOR CONTRIBUTIONS

M.T., N.H., M. T., and K.S. contributed to the conception and design of the study. M.T., N.I., K.H., K.H., and R.M. contributed to the acquisition of data. M.T., K.A., A.W., and K.S. contributed to the analysis and interpretation of data. M.T., N.H., and K.S. contributed to the drafting of the manuscript. M.T., N.H., M.T., and K.S. contributed to the critical revision of the manuscript. All authors contributed to the final approval of the manuscript.

## FUNDING INFORMATION

This research did not receive any specific grant from funding agencies in the public, commercial, or not‐for‐profit sectors.

## CONFLICT OF INTEREST STATEMENT

Author Ken Shirabe is an editorial board member of *Annals of Gastroenterological Surgery*. The other authors declare no conflict of interest for this article. The authors report no proprietary or commercial interest in any product mentioned or concept discussed in this article.

## ETHICS STATEMENT

The study was approved by the Ethics Committee of the study hospital (HS2019‐306) and was conducted in accordance with the institutional guidelines and the Declaration of Helsinki. Patient consent for participation was obtained using the opt‐out method.

## References

[1] Siegel RL , Miller KD , Fuchs HE , Jemal A . Cancer statistics, 2022. CA Cancer J Clin. 2022;72(1):7–33. 35020204 10.3322/caac.21708

[2] Yamaue H . History of pancreatic surgery in Japan: respect to the Japanese pioneers of pancreatic surgery. Ann Gastroenterol Surg. 2020;4(2):118–25. 32258976 10.1002/ags3.12320 PMC7105840

[3] Bundred J , Kamarajah SK , Roberts KJ . Body composition assessment and sarcopenia in patients with pancreatic cancer: a systematic review and meta‐analysis. HPB (Oxford). 2019;21(12):1603–12. 31266698 10.1016/j.hpb.2019.05.018

[4] Tsukagoshi M , Harimoto N , Araki K , Kubo N , Watanabe A , Igarashi T , et al. Skeletal muscle loss and octogenarian status are associated with S‐1 adjuvant therapy discontinuation and poor prognosis after pancreatectomy. Cancers (Basel). 2021;13(16):4105. 34439259 10.3390/cancers13164105 PMC8391507

[5] Cruz‐Jentoft AJ , Bahat G , Bauer J , Boirie Y , Bruyère O , Cederholm T , et al. Sarcopenia: revised European consensus on definition and diagnosis. Age Ageing. 2019;48:16–31. 30312372 10.1093/ageing/afy169 PMC6322506

[6] Levey AS , Perrone RD , Madias NE . Serum creatinine and renal function. Annu Rev Med. 1988;39:465–90. 3285786 10.1146/annurev.me.39.020188.002341

[7] Thongprayoon C , Cheungpasitporn W , Kashani K . Serum creatinine level, a surrogate of muscle mass, predicts mortality in critically ill patients. J Thorac Dis. 2016;8(5):E305–11. 27162688 10.21037/jtd.2016.03.62 PMC4842835

[8] Dharnidharka VR , Kwon C , Stevens G . Serum cystatin C is superior to serum creatinine as a marker of kidney function: a meta‐analysis. Am J Kidney Dis. 2002;40(2):221–6. 12148093 10.1053/ajkd.2002.34487

[9] Osaka T , Hamaguchi M , Hashimoto Y , Ushigome E , Tanaka M , Yamazaki M , et al. Decreased the creatinine to cystatin C ratio is a surrogate marker of sarcopenia in patients with type 2 diabetes. Diabetes Res Clin Pract. 2018;139:52–8. 29496508 10.1016/j.diabres.2018.02.025

[10] Lin YL , Chen SY , Lai YH , Wang CH , Kuo CH , Liou HH , et al. Serum creatinine to cystatin C ratio predicts skeletal muscle mass and strength in patients with non‐dialysis chronic kidney disease. Clin Nutr. 2020;39(8):2435–41. 31732290 10.1016/j.clnu.2019.10.027

[11] Tabara Y , Kohara K , Okada Y , Ohyagi Y , Igase M . Creatinine‐to‐cystatin C ratio as a marker of skeletal muscle mass in older adults: J‐SHIPP study. Clin Nutr. 2020;39(6):1857–62. 31431305 10.1016/j.clnu.2019.07.027

[12] Zheng C , Wang E , Li JS , Xie K , Luo C , Ge QY , et al. Serum creatinine/cystatin C ratio as a screening tool for sarcopenia and prognostic indicator for patients with esophageal cancer. BMC Geriatr. 2022;22(1):207. 35287579 10.1186/s12877-022-02925-8 PMC8922862

[13] Sun J , Yang H , Cai W , Zheng J , Shen N , Yang X , et al. Serum creatinine/cystatin C ratio as a surrogate marker for sarcopenia in patients with gastric cancer. BMC Gastroenterol. 2022;22(1):26. 35045814 10.1186/s12876-022-02093-4 PMC8772102

[14] Dindo D , Demartines N , Clavien PA . Classification of surgical complications: a new proposal with evaluation in a cohort of 6336 patients and results of a survey. Ann Surg. 2004;240(2):205–13. 15273542 10.1097/01.sla.0000133083.54934.ae PMC1360123

[15] Nishikawa H , Shiraki M , Hiramatsu A , Moriya K , Hino K , Nishiguchi S . Japan Society of Hepatology guidelines for sarcopenia in liver disease (1st edition): recommendation from the working group for creation of sarcopenia assessment criteria. Hepatol Res. 2016;46(10):951–63. 27481650 10.1111/hepr.12774

[16] Auyeung TW , Arai H , Chen LK , Woo J . Letter to the editor: normative data of handgrip strength in 26344 older adults ‐ a pooled dataset from eight cohorts in Asia. J Nutr Health Aging. 2020;24(1):125–6.3188681910.1007/s12603-019-1287-6

[17] Chan AW , Chan SL , Wong GL , Wong VW , Chong CC , Lai PB , et al. Prognostic nutritional index (PNI) predicts tumor recurrence of very early/early stage hepatocellular carcinoma after surgical resection. Ann Surg Oncol. 2015;22(13):4138–48. 25801356 10.1245/s10434-015-4516-1

[18] Hilmi M , Jouinot A , Burns R , Pigneur F , Mounier R , Gondin J , et al. Body composition and sarcopenia: the next‐generation of personalized oncology and pharmacology? Pharmacol Ther. 2019;196:135–59. 30521882 10.1016/j.pharmthera.2018.12.003

[19] Tsukagoshi M , Harimoto N , Araki K , Kubo N , Watanabe A , Igarashi T , et al. Impact of preoperative nutritional support and rehabilitation therapy in patients undergoing pancreaticoduodenectomy. Int J Clin Oncol. 2021;26(9):1698–706. 34089094 10.1007/s10147-021-01958-0

[20] Kabasawa K , Nakamura K , Ito Y , Tanaka J , Narita I . Association between estimated glomerular filtration rate based on cystatin C and grip strength in community‐dwelling Japanese older adults. J Gerontol A Biol Sci Med Sci. 2021;76(9):1653–60. 32971533 10.1093/gerona/glaa240

[21] Kim SW , Jung HW , Kim CH , Kim KI , Chin HJ , Lee H . A new equation to estimate muscle mass from creatinine and cystatin C. PLoS One. 2016;11(2):e0148495. 26849842 10.1371/journal.pone.0148495 PMC4744004

[22] Kashani KB , Frazee EN , Kukrálová L , Sarvottam K , Herasevich V , Young PM , et al. Evaluating muscle mass by using markers of kidney function: development of the sarcopenia index. Crit Care Med. 2017;45(1):e23–9. 27611976 10.1097/CCM.0000000000002013

[23] Yang J , Zhang T , Feng D , Dai X , Lv T , Wang X , et al. A new diagnostic index for sarcopenia and its association with short‐term postoperative complications in patients undergoing surgery for colorectal cancer. Color Dis. 2019;21(5):538–47. 10.1111/codi.14558 30648340

[24] Ulmann G , Kai J , Durand JP , Neveux N , Jouinot A , De Bandt JP , et al. Creatinine‐to‐cystatin C ratio and bioelectrical impedance analysis for the assessement of low lean body mass in cancer patients: comparison to L3‐computed tomography scan. Nutrition. 2021;81:110895. 32739656 10.1016/j.nut.2020.110895

[25] Tang T , Xie L , Hu S , Tan L , Lei X , Luo X , et al. Serum creatinine and cystatin C‐based diagnostic indices for sarcopenia in advanced non‐small cell lung cancer. J Cachexia Sarcopenia Muscle. 2022;13(3):1800–10. 35297568 10.1002/jcsm.12977 PMC9178169

[26] Gowda S , Desai PB , Kulkarni SS , Hull VV , Math AA , Vernekar SN . Markers of renal function tests. N Am J Med Sci. 2010;2(4):170–3. 22624135 PMC3354405

[27] Fu X , Tian Z , Wen S , Sun H , Thapa S , Xiong H , et al. A new index based on serum creatinine and cystatin C is useful for assessing sarcopenia in patients with advanced cancer. Nutrition. 2021;82:111032. 33172686 10.1016/j.nut.2020.111032

[28] Jung CY , Kim H , Han S , Yoo TH , Kang SW , Park JT . Creatinine‐cystatin C ratio and mortality in cancer patients: a retrospective cohort study. J Cachexia Sarcopenia Muscle. 2022;13(4):2064–72. 35478277 10.1002/jcsm.13006 PMC9397493

[29] Gao J , Liang H , Qian Y , Pan J , Liu W , Qi W , et al. Creatinine‐to‐cystatin C ratio as a marker of skeletal muscle mass for predicting postoperative complications in patients undergoing gastric cancer surgery. Ann Palliat Med. 2021;10(5):5017–26. 33894713 10.21037/apm-20-2366

[30] Tufan A , Tufan F , Akpinar TS , Ilhan B , Bahat G , Karan MA . Low glomerular filtration rate as an associated risk factor for sarcopenic muscle strength: is creatinine or cystatin C‐based estimation more relevant? Aging Male. 2017;20(2):110–4. 27650550 10.1080/13685538.2016.1225032

[31] Harimoto N , Araki K , Yamanaka T , Hagiwara K , Ishii N , Tsukagoshi M , et al. The ratio of creatinine and cystatin C estimated glomerular filtration rates as a surrogate marker in patients with hepatocellular carcinoma undergoing hepatic resection. J Hepatobiliary Pancreat Sci. 2022;29:964–73. 35543073 10.1002/jhbp.1164

